# Evaluating Neck Circumference as an Independent Predictor of Metabolic Syndrome and Its Components Among Adults: A Population-Based Study

**DOI:** 10.7759/cureus.40379

**Published:** 2023-06-13

**Authors:** Sahar Mohseni-Takalloo, Hassan Mozaffari-Khosravi, Hadis Mohseni, Masoud Mirzaei, Mahdieh Hosseinzadeh

**Affiliations:** 1 Nutrition and Food Security Research Center, Shahid Sadoughi University of Medical Sciences, Yazd, IRN; 2 Department of Nutrition, School of Public Health, Shahid Sadoughi University of Medical Sciences, Yazd, IRN; 3 School of Public Health, Bam University of Medical Sciences, Bam, IRN; 4 Computer Engineering Department, Shahid Bahonar University of Kerman, Kerman, IRN; 5 Yazd Cardiovascular Research Centre, Non-communicable Diseases Research Institute, Shahid Sadoughi University of Medical Sciences, Yazd, IRN

**Keywords:** dyslipidemia, hyperglycemia, blood pressure, obesity, neck circumference, metabolic syndrome

## Abstract

Background

Metabolic syndrome (MetS), a public health problem worldwide, represents a high-risk condition for cardiovascular disease and diabetes. The reserve of subcutaneous fat in the upper torso is an important factor in the development of MetS and its risk factors. As neck circumference (NC) is a simple and reliable index that indicates upper-body subcutaneous fat accumulation, this study was conducted to investigate the association between NC with MetS and its components in a large population-based sample of Iranian adults.

Methods

The present cross-sectional study was conducted among 2094 individuals aged 20-70 years from Yazd Health Study (YaHS). MetS was defined based on the National Cholesterol Education Program Adult Treatment Panel III (ATP III) criteria. NC more than 40.25 cm for men and more than 35.75 cm for women was considered a high-risk NC. Logistic regression analysis was applied to obtain the associations of NC with MetS and its component.

Results

After adjusting for age, BMI, energy intake, physical activity, and smoking in those whose NC was higher than normal, the risk of the MetS (odds ratio {OR}: 2.32; 95%CI: 1.55-3.46, P<0.001 in men and OR: 2.56; 95%CI: 1.76-3.74, P<0.001 in women), abdominal obesity (OR: 4.39; 95%CI: 2.67-7.23, P<0.001 in men and OR: 1.92; 95%CI: 1.27-2.90, P=0.002 in women), high blood pressure (OR: 1.54; 95%CI: 1.07-2.21, P=0.02 in men and OR: 1.51; 95%CI: 1.06-2.14, P=0.02 in women), low high-density lipoprotein cholesterol (HDL-C) (OR: 1.47; 95%CI: 1.01-2.15, P=0.04 in men and OR: 1.69; 95%CI: 1.23-2.32, P=0.001 in women), and hypertriglyceridemia (OR: 1.41; 95%CI: 1.03-1.99, P=0.04 in men and OR: 1.68; 95%CI: 1.17-2.41, P=0.005 in women) were higher. There was no significant difference in the risk of hyperglycemia between the two NC groups in both sexes. The Pearson's correlation coefficients of NC with waist circumference, hip circumference, body mass index, and waist-to-height ratio were 0.52, 0.43, 0.41, and 0.31, respectively (P<0.001). Moreover, NC had a considerable correlation with serum triglyceride, high-density lipoprotein, systolic blood pressure, and diastolic blood pressure (0.27, -0.30, 0.29, 0.25, P<0.001), respectively.

Conclusion

Increased NC was significantly associated with higher odds of MetS and its components. Since NC measurement is simple, inexpensive, reliable, and less invasive, it can be used as a complementary tool in the screening and diagnosis of MetS and its risk factors in clinical and community programs especially in developing countries.

## Introduction

A metabolic syndrome (MetS) is characterized by a combination of metabolic disorders including abdominal obesity, hyperglycemia, dyslipidemia, and elevated blood pressure, indicating a high-risk condition for cardiovascular disease, type 2 diabetes, and fatty liver [1]. These diseases are a major cause of disability, premature death, and the economic burden of health care worldwide [2]. According to lifestyle changes and nutritional transition, the prevalence of MetS is increasing in parallel with the rise in obesity rates in developing countries. Therefore, early detection of MetS risk is crucial [3].

The fat distribution in the upper torso is the most important factor in MetS and cardiovascular disease development. Waist circumference is an indicator of abdominal obesity and visceral fat. However, its measurement may include errors depending on factors such as waist circumference anatomic measurement location, pendulous abdomen after significant weight loss, severe obesity, before meal versus after meal measurement, heavy clothing, and body standing position. In addition, in societies where women wear hijabs, like Middle-eastern countries, there are cultural barriers to removing the upper body clothing for more accurate waist measurement [4].

Neck Circumference (NC) is a simple, inexpensive, and reliable alternative for waist circumference that is not affected by the mentioned limitations [5]. It has been shown that NC indicates upper-body subcutaneous fat accumulation and is a reliable predictive tool for abdominal and overall obesity [6,7]. NC has been shown to correlate with markers of cardio-metabolic risk, hypertension, insulin resistance, and MetS. In Brazil, a population-based study positively associated NC with triglycerides, insulin resistance, blood pressure, body mass index, and total body fat [8]. Other studies have also correlated NC with hypertension [9,10], insulin resistance [11], and cardio-metabolic risk factors [12,13]. In severe obesity, NC performance might be better than waist circumference in assessing metabolic health [14]. In Korea and Thailand, NC was recognized as a predictor of MetS in adults [15,16]. Moreover, it has been shown that NC predicts metabolic syndrome and obesity in Bangladeshi women with polycystic ovary syndrome [17]. Since the predictive potential of NC for MetS and its components has varied among different ethnic populations [18], and the prevalence of MetS in Iran, like in other developing countries, is high (about 30%) [19], and also NC measurement is simple, inexpensive, and less invasive, therefore, this study aimed to investigate the association between NC with MetS and its components in a large population-based sample of Iranian adults.

## Materials and methods

Study design and participants

The present cross-sectional study was conducted on data from the recruitment phase of a population-based cohort study known as Yazd Health Study (YaHS), Iran (www.yahs-ziba.com). Detailed information about the methodology and profile of the study was published elsewhere [20]. In brief, 9962 individuals aged 20-70 years from the urban areas of Yazd, in 2014-2015 entered the study to determine the prevalence of a variety of chronic diseases and associated risk factors. Subjects were excluded from this study if (1) their demographic, anthropometric, biochemical, or blood pressure data were not complete, (2) they suffered from chronic diseases (including cardiovascular disease, diabetes mellitus, hypertension, fatty liver, thyroid disease, and different types of cancer), (3) were pregnant or breastfeeding. Finally, a total of 2094 individuals (1066 male and 1028 female) were eligible for further analysis. Informed consent was obtained from all participants and the present study has been approved by the Ethics Committee at Shahid Sadoughi University of Medical Sciences (approval code: IR.SSU.SPH.REC.1399.202).

Anthropometric measurements

Anthropometric data were collected by trained research staff. Body weight was measured using an Omron BF511 digital scale (with an accuracy of 100 grams) (Omron Inc., Nagoya, Japan), while individuals wore minimum clothing. Height was measured in a standing position without shoes using non-stretchable tape on a wall with 1-centimeter scale precision. BMI was obtained as the weight in kilograms (kg) divided by the square of height in meters (m^2^). Waist circumference and hip circumference were recorded in the standing position using rigid tape placed midpoint of the iliac crest and the lowest rib, and over the maximum extension of the buttocks, respectively, with an accuracy of 0.5 cm. The NC was measured around the base of the neck below the thyroid nearest 0.5 cm, using an unstretched tape, while it was kept slightly loose and not restricted on the neck [18]. Based on previous research on the present papulation, NC above 40.25 cm for men and above 35.75 cm for women indicates a high risk for obesity [21]. This cut-off point was used to classify the participants into high-risk and low-risk groups.

Laboratory and blood pressure measurements

Laboratory measurements (including glucose and lipid concentrations) were obtained from at least eight hours of fasting blood samples according to a standard laboratory protocol using Pars Azmoon kits (Pars Azmoon Inc., Tehran, Iran) and calibrated Ciba Corning auto-analyzers (Ciba Corp., Basle, Switzerland). Systolic and diastolic blood pressures were measured after at least 40-minutes of rest, thrice in five-minute intervals by Reichter electronic sphygmomanometers (Model N-Champion, Reister GMBH, Germany), and the mean of record was considered as the individual's blood pressure [20].

Metabolic syndrome definitions

MetS was defined based on the guidelines of the National Cholesterol Education Program Adult Treatment Panel III (ATP III) so that having three of the following criteria indicates MetS: (1) fasting blood glucose ≥100 mg/dl; (2) serum triglycerides ≥150 mg/dl; (3) serum high-density lipoprotein cholesterol (HDL-C) < 50 mg/dl in women and <40 mg/dl in men; (4) waist circumference >88 cm in women and >102 cm in men; and (5) systolic blood pressure ≥130 mmHg and/or diastolic blood pressure ≥85 mmHg.

Statistical analysis

Statistical analyses were performed using IBM SPSS Statistic software version 22.0 (IBM Corp., Armonk, NY), and a P value lower than 0.05 was considered as the significance level. Descriptive statistics were expressed for continuous variables as mean ± standard deviation (SD) using two independent t-tests, and categorical variables as frequencies (percentages) using chi-square. Logistic regression analysis was applied to obtain the risk of MetS and its component based on NC categories while adjusting for age, BMI, energy intake, physical activity, and smoking. The matrix of Pearson's correlation coefficient between anthropometric and biochemical variables was drawn with the Python software package incorporated in the Anaconda Navigator (version 1.00; Anaconda Software Distribution, www.anaconda.com).

## Results

Among the 2094 participants, 50.9% were men and 49.1% were women, with a mean age of 53.0±17.7 and 50.9±16.8 years, respectively. The demographic, anthropometric, and biochemical characteristics of the study population, according to sex, are shown in Table 1. The MetS was significantly more prevalent in females than in males (33.3 vs 23.2). Waist circumference, neck circumference, triglycerides, and systolic and diastolic blood pressure were higher in males compared to females (p<0.001), while BMI, hip circumference, and HDL-C were lower in males than in females (p<0.001). No significant differences were observed in fasting blood glucose, total cholesterol, and LDL-C concentrations between the two sexes.

**Table 1:** Demographic, anthropometric, and biochemical characteristics of the recruitment phase of Yazd Health Study participants by sex (2014-2015) BMI: Body Mass Index, HDL-C: High-density lipoprotein cholesterol, LDL-C: low-density lipoprotein cholesterol

	Male (n=1066)	Female (n=1028)	Total (n=2094)	
Parameters	Mean±SD	Mean±SD	Mean±SD	P_ value_
Age (years)	53.0±17.7	50.9±16.8	52.0±17.3	0.005
BMI (kg/m2)	26.0±4.6	27.1±5.3	26.6±5.0	<0.001
Waist circumference (cm)	92.6±12.7	90.6±12.8	91.6±12.8	<0.001
Hip circumference (cm)	99.3±10.7	101.8±11.8	100.6±11.3	<0.001
Neck circumference (cm)	38.9±3.2	35.4±3.1	37.2±3.6	<0.001
Fasting blood glucose (mg/dL)	96.2±18.1	96.2±19.3	96.3±18.9	0.93
Triglycerides (mg/dL)	160.7±102.9	129.6±76.7	145.4±92.2	<0.001
Total cholesterol (mg/dL)	190.3±39.5	192.5±39.6	191.4±39.6	0.24
HDL-C (mg/dL)	45.8±8.8	52.8±11.3	49.2±10.7	<0.001
LDL-C (mg/dL)	112.4±34.7	113.7±33.2	113.1±34.0	0.42
Systolic blood pressure (mmHg)	125.5±13.9	120.0±15.6	122.8±15.0	<0.001
Diastolic blood pressure (mmHg)	80.0±10.1	76.9±10.2	78.5±10.2	<0.001
	n (%)	n (%)	n (%)	
Metabolic syndrome (Yes)	247 (23.2)	342 (33.3)	589 (28.2)	<0.001

**Figure 1:**
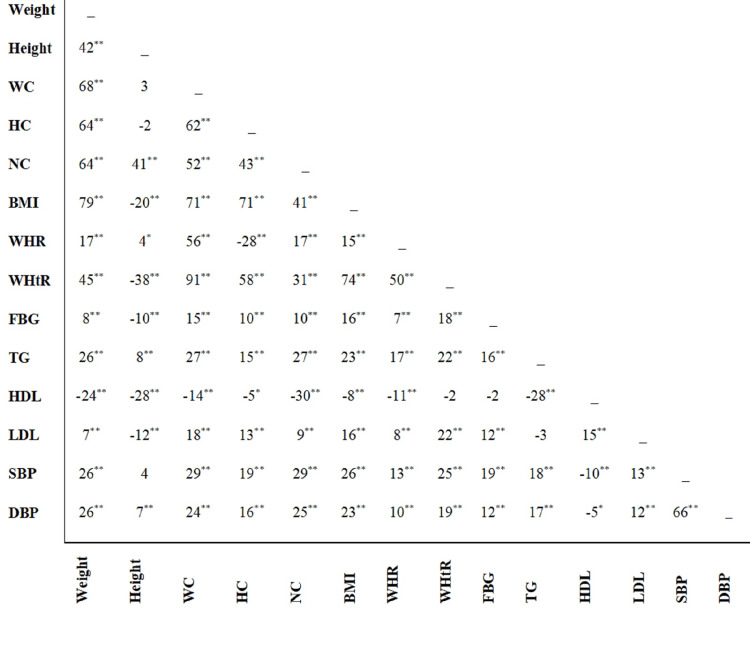
Pearson's correlation coefficient of anthropometric, and biochemical characteristics of the recruitment phase of Yazd Health Study participants ** P value<0.01, * P value<0.05 WC: waist circumference, HC: hip circumference, NC: neck circumference, BMI: Body Mass Index, WHR: waist to hip ratio, WHtR: waist to height ratio, FBG: fasting blood glucose, TG: triglycerides, HDL-C: high-density lipoprotein cholesterol, LDL-C: low-density lipoprotein cholesterol, SBP: systolic blood pressure, DBP: diastolic blood pressure

The Pearson's correlation coefficient matrix of the anthropometric, and biochemical characteristics of participants is displayed in Figure 1. Among the anthropometric indices, NC has the highest correlation with serum HDL-C concentration (r=-0.30). Also, waist circumference and NC are most closely related to serum triglyceride concentration (r=0.27) and systolic blood pressure (r=0.29). The correlation of NC with waist circumference and BMI is equal to 0.52 and 0.41, respectively.

Table 2 demonstrates that the MetS and all of its components were more frequent in the high-risk NC group in both sexes.

**Table 2:** The percent of MetS and its components according to NC categories by sex MetS: Metabolic syndrome; HDL-C: High-density lipoprotein cholesterol

MetS and its components	Male			Female			Total		
	Low risk <=40.25	High risk >40.25	P value	Low risk <=35.75	High risk >35.75	P value	Low risk <=40.25/ <=35.75	High risk >40.25/ >35.75	Pvalue
Metabolic syndrome	14.6	45.2	<0.001	19.9	49.8	<0.001	16.9	48.0	<0.001
Abdominal obesity	10.1	51.5	<0.001	44.6	77.6	<0.001	24.8	67.3	<0.001
High blood pressure	42.6	60.2	<0.001	24.5	39.8	<0.001	34.9	47.8	<0.001
Hyperglycemia	23.1	35.1	<0.001	22.9	33.5	<0.001	23.0	34.1	<0.001
Low HDL-C	21.1	33.1	<0.001	35.6	49.3	<0.001	27.3	43.0	<0.001
Hypertriglyceridemia	36.2	54.5	<0.001	21.0	30.8	<0.001	29.7	45.6	<0.001

The odds ratio of MetS components in the high-risk NC group is indicated in Table 3. It was found that after adjustment for age, BMI, energy intake, physical activity, and smoking, the risks of abdominal obesity, high blood pressure, hyperglycemia, low HDL-C, and hypertriglyceridemia were 4.39 (95% CI: 2.67-7.23, P<0.001), 1.54 (95% CI: 1.07-2.21, P=0.02), 1.33 (95% CI: 0.91-1.95, P=0.14), 1.47 (95% CI: 1.01-2.15, P=0.04), 1.41 (95% CI: 1.03-1.99, P=0.04) in the males with high-risk NC, and 1.92 (95% CI: 1.27-2.90, P=0.002), 1.51 (95% CI: 1.06-2.14, P=0.02), 1.14 (95% CI: 0.80-1.63, P=0.47), 1.69 (95% CI: 1.23-2.32, P=0.001), and 1.68 (95% CI: 1.17-2.41, P=0.005) in the females with high-risk NC, respectively.

**Table 3:** Risk of MetS components in the high-risk group of neck circumference *Models adjusted for age, BMI, energy intake, physical activity, and smoking MetS: Metabolic syndrome; HDL-C: high-density lipoprotein cholesterol

MetS components	Male		Female		Total	
	OR (95% CI)	P value*	OR (95% CI)	P value*	OR (95% CI)	P value*
Abdominal obesity	4.39 (2.67-7.23)	<0.001	1.92 (1.27-2.90)	0.002	2.76 (2.09-3.66)	<0.001
High blood pressure	1.54 (1.07-2.21)	0.02	1.51 (1.06-2.14)	0.02	1.29 (1.02-1.63)	0.04
Hyperglycemia	1.33 (0.91-1.95)	0.14	1.14 (0.80-1.63)	0.47	1.23 (0.95-1.59)	0.11
Low HDL-C	1.47 (1.01-2.15)	0.04	1.69 (1.23-2.32)	0.001	1.74 (1.37-2.21)	<0.001
Hypertriglyceridemia	1.41 (1.03-1.99)	0.04	1.68 (1.17-2.41)	0.005	1.33 (1.04-1.69)	0.02

The odds ratio of MetS after adjusting for age, BMI, energy intake, physical activity, and smoking in the NC high-risk group compared to the NC low-risk group were 2.32 (95% CI: 1.55-3.46, P<0.001) in males and 2.56 (95% CI: 1.76-3.74, P<0.001) in females (Table 4).

**Table 4:** Risk of MetS in the high-risk group of neck circumference *Model 1: crud, **Model 2: adjusted for age and BMI, ***Model 3: adjusted for age, BMI, energy intake, physical activity, and smoking MetS: Metabolic syndrome

MetS prediction models	Male		Female		Total	
	OR (95% CI)	P value	OR (95% CI)	P value	OR (95% CI)	P value
Model 1^*^	4.81 (3.55-6.51)	<0.001	3.98 (3.02-5.24)	<0.001	4.54 (3.71-5.55)	<0.001
Model 2^**^	2.86 (2.02-4.04)	<0.001	2.51 (1.81-3.46)	<0.001	2.72 (2.15-3.45)	<0.001
Model 3^***^	2.32 (1.55-3.46)	<0.001	2.56 (1.76-3.74)	<0.001	2.49 (1.90-3.26)	<0.001

## Discussion

This population-based study revealed that NC could be a potentially powerful tool for the prediction and screening of MetS. It was found that after adjustment for age and BMI, energy intake, physical activity, and smoking, the risk of MetS in males and females with high-risk NC is 2.32 and 2.56 times higher versus those with low-risk NC, respectively. In both sexes, those with high-risk NC had a greater risk for MetS components than those with low-risk NC, except hyperglycemia. Also, NC strongly correlated with the obesity anthropometric markers as well as with blood pressure, and MetS biochemical parameters.

The results of the present study were in line with most of the previous investigations. A study conducted in Korea found that after adjustment for age, history of diseases, family history of diseases, smoking, intakes of energy, sugar, and sodium, BMI, and waist circumference, the risk of MetS in high-risk NC compared to the low-risk NC was 2.01 and 3.65 fold in males and females, respectively [15]. Meanwhile, in Saudi adults, after adjustment for age and waist-to-height ratio, the odds of MetS in males and females with high-risk NC were 3.03 and 3.66 times higher than those with optimal NC [4]. In a research carried out in Thailand, the NC was a useful tool for predicting MetS and its component in both sexes, except for low HDL-C and hyperglycemia in men [16]. Among Pakistani adults, the odds of metabolic syndrome, abdominal obesity, and hyperglycemia in both sexes and the odds of hypertriglyceridemia and low HDL just in men were significantly higher in subjects with high-risk NC [5]. However, in Indian adults, the prevalence of metabolic syndrome and all of its components were higher in those with high-risk NC [22]. Also, in Bangladeshi women with polycystic ovary syndrome, the metabolic syndrome and its components, except fasting blood glucose and HDL-C, were significantly increased across the NC quartiles [17]. NC was significantly correlated with cardio-metabolic risk factors including systolic blood pressure and diastolic blood pressure, fasting blood glucose, triglyceride, LDL-C, and HDL-C, in China. The relationship between total cholesterol and NC was observed only in men [23]. However, a study in Lebanon showed that NC was only associated with LDL-C and triglyceride in males and LDL-C, total cholesterol, and systolic blood pressure in females [24]. Differences observed in studies results could be due to racial and geographic disparities in populations, the method used to measure NC in particular research, and risk factors that have been adjusted in the analyses [24].

Consistent with our results, previous studies have shown that NC has a strong correlation with BMI, waist circumference, and waist-to-height ratio, suggesting that NC can be considered an indicator of obesity [7,15,16,25]. Since central obesity is measured by waist circumference, one of the main components in the diagnosis of MetS, its correlation with NC is of great importance. Waist circumference is an accepted tool for assessing abdominal obesity. However, its assessment is accompanied by some limitations such that breathing and abdominal expansion after meals or bloating affect its accurate measurement. Also, in pregnant women and people with hernia or ascites, waist circumference cannot be considered an indicator of abdominal obesity [15]. These limitations show the necessity of using other anthropometric indicators besides waist circumference. It has been reported that combining NC with obesity anthropometric measurements (e.g. BMI and waist circumference) can improve disease risk prediction [26]. NC measurement does not have the limitations of waist circumference measurement; it is convenient, reliable, and does not require undressing. This can be helpful in hijab-wearing communities or people with body image issues [24]. However, the measurement of NC in individuals with thyroid disease and neck masses is limited [27]. It has been indicated that in morbid obesity, NC can better reflect metabolic health than waist circumference [14]. By measuring NC, it is possible to estimate upper-body subcutaneous fat, which has a significant relationship with MetS and cardiovascular risk factors [28]. The amount of upper-body subcutaneous fat is one of the main determining factors in serum free-fatty acids concentration. Increased concentration of free fatty acids is associated with insulin resistance and endothelial cell dysfunction [29]. In addition, in adults, brown adipose tissue which is mainly located in the neck area and around the carotid arteries, plays an important role in energy homeostasis and adipokines secretion such as interleukin-6 and leptin. The activity of this tissue decreases with increasing excess weight, resulting in metabolic disorders [18]. In this way, the relationship between NC and metabolic diseases can be partially explained. Also, an abnormal NC in some individuals without MetS can be due to the presence of only one or two components of MetS in these people, and the three criteria required for a diagnosis of MetS are not met [22].

The present study had several strengths: first, investigators were fully trained to perform anthropometric measurements before starting the study. Second, The study sample size was relatively large (2094 people) and included both sexes. However, there are some limitations; the cross-sectional design of the study limits the inference of causal relationships. Also, considering that the NC cutoff was determined based on the YaHS population, the results may not be generalized to other ethnic or racial groups.

## Conclusions

In conclusion, NC, a new index for obesity assessment, has a significant association with MetS and its components, as well as obesity anthropometric indicators. NC measurement is simple, inexpensive, accessible, and less invasive. Therefore, its use as a complementary tool in the screening and diagnosis of MetS and its risk factors is justified in both clinical and community settings so that patients with abnormal NC should be screened for MetS risk factors. Further, longitudinal studies are needed to confirm the results of this study and to determine whether NC alone or in combination with other obesity indices is better for predicting MetS and its risk factors.
